# Utility of Insulin Resistance in Estimating Cardiovascular Risk in Subjects with Type 1 Diabetes According to the Scores of the Steno Type 1 Risk Engine

**DOI:** 10.3390/jcm9072192

**Published:** 2020-07-11

**Authors:** Albert Cano, Gemma Llauradó, Lara Albert, Isabel Mazarico, Brenno Astiarraga, Montserrat González-Sastre, Laia Martínez, Sonia Fernández-Veledo, Rafael Simó, Joan Vendrell, José-Miguel González-Clemente

**Affiliations:** 1 Department of Endocrinology and Nutrition, Parc Taulí Hospital Universitari, Institut d’Investigació i Innovació Parc Taulí I3PT, Universitat Autònoma de Barcelona, Parc Taulí 1, 08208 Sabadell, Spain; ACANO@tauli.cat (A.C.); LAlbert@tauli.cat (L.A.); imazarico@tauli.cat (I.M.); 2 Department of Endocrinology and Nutrition, Hospital del Mar, Institut Hospital del Mar d’Investigacions Mèdiques (IMIM), Universitat Autònoma de Barcelona, Pg. Marítim 25-29, 08003 Barcelona, Spain; gllauradoc@gmail.com; 3 Institut d’Investigacions Sanitàries Pere Virgili (IISPV), Universitat Rovira i Virgili, Avda. de la Universitat, 43204 Reus, Spain; bdastiarraga@gmail.com (B.A.); sonia.fernandezveledo@gmail.com (S.F.-V.); jvortega2002@gmail.com (J.V.); 4 Centro de Investigación Biomédica en Red de Diabetes y Enfermedades Metabólicas Asociadas (CIBERDEM), Instituto de Salud Carlos III, 08029 Madrid, Spain; rafael.simo@vhir.org; 5 Department of Endocrinology and Nutrition, Hospital Universitari Joan XXIII de Tarragona, IISPV, Universitat Rovira i Virgili, C. Dr Mallafré Guasch 4, 43005 Tarragona, Spain; laiamguasch@gmail.com; 6 Ophthalmology Department, Parc Taulí Hospital Universitari, Institut d’Investigació i Innovació Parc Taulí I3PT, Universitat Autònoma de Barcelona, Parc Taulí 1, 08208 Sabadell, Spain; montse_gonzalez_sastre@hotmail.com; 7 Diabetes and Metabolism Research Unit, Institut de Recerca Hospital Universitari Vall d’Hebron, Universitat Autònoma de Barcelona, Pg. de la Vall d’Hebron, 119-129, 08035 Barcelona, Spain

**Keywords:** type 1 diabetes, cardiovascular risk, steno type 1 risk engine, estimated insulin sensitivity, insulin resistance

## Abstract

Background: We sought to assess the potential of insulin resistance (IR) for estimating cardiovascular disease (CVD) risk in adults with type 1 diabetes (T1DM) according to the scores of the Steno Type 1 Risk Engine (ST1RE). Methods: A total of 179 adults with T1DM (50.8% men, age 41.2 ± 13.1 years, duration of T1DM 16 (12–23) years) without established CVD were evaluated. IR was assessed by the estimation of insulin sensitivity (eIS) using two validated prediction equations: the estimated insulin sensitivity developed from the Pittsburgh Epidemiology of Diabetes Complications Study (eIS-EDC) and the estimated insulin sensitivity developed from Coronary Artery Calcification in T1DM Study (eIS-CACTI) ST1RE was used to estimate 10-year CVD risk and to classify subjects into three groups according to their risk: low (<10%; *n* = 105), moderate (10–20%; *n* = 53), and high (≥20%; *n* = 21). Results: Both eIS-EDC and eIS-CACTI correlated negatively with ST1RE scores (eIS-EDC: r = −0.636, *p* < 0.001; eIS-CACTI: r = −0.291, *p* < 0.001). The C-statistic for predicting moderate/high risk and high risk was 0.816 (95% confidence interval (CI): 0.754–0.878) and 0.843 (95% CI: 0.772–0.913), respectively, for the eIS-EDC equation, and was 0.686 (95% CI: 0.609–0.763) and 0.646 (95% CI: 0.513–0.778), respectively, for the eIS-CACTI equation. The eIS-EDC equation had a significantly higher C-statistic both for moderate-/high-risk (*p* = 0.001) and high-risk (*p* = 0.007) subjects. Two cut-off points of eIS-EDC were identified for detecting moderate/high risk (8.52 mg·kg^−1^·min^−1^; sensitivity 74% and specificity 76%) and high risk (8.08 mg·kg^−1^·min^−1^; sensitivity 65% and specificity 95%) with potential applicability in clinical practice. Conclusions: eIS negatively correlates with the score of CVD risk in the ST1RE. Two cut-off points of eIS are reported with potential utility in clinical practice for detecting adults with T1DM with the highest CVD risk.

## 1. Introduction

Type 1 diabetes mellitus (T1DM) remains a serious chronic disorder with an estimated life-expectancy loss of about 11 years in men and 13 years in women, mainly due to cardiovascular disease (CVD), with coronary artery disease (CAD) representing up to one-third of this loss [1]. Indeed, CVD is the leading cause of death in people with T1DM, with an estimated relative incidence 2–8 times higher than that reported in people without the condition [2,3,4,5]. While recent epidemiological studies have reported lower relative risk of CVD and CAD than previously described [4], CVD risk remains unacceptably high for people with T1DM [6,7], and simple and easy-to-use scores for accurately classifying people with T1DM according to their estimated CVD risk are still urgently needed as a first step for decreasing CVD burden in T1DM.

Several risk scores for estimating CVD risk have been developed both in the general population (e.g., Framingham Risk Score) [8] and in people with type 2 diabetes (United Kingdom Prospective Diabetes Study-UKPDS-Risk Engine) [9], but these scores clearly underestimate CVD risk in T1DM [10]. In the last 10 years, three risk scores have been specifically developed for estimating CVD risk in people with T1DM. The first score was initially developed in the Pittsburgh Epidemiology of Diabetes Complications (EDC) study, and was aimed to estimate CAD risk [11]. The second one, from the Swedish National Diabetes Register, was developed in people with T1DM with or without previous CVD and estimated 5-year CVD risk [12]. The third risk score was developed in a large cohort of the Steno Diabetes Center for estimating 10-year CVD risk using the Steno Type 1 Risk Engine (ST1RE) [13]. Of note, the ST1RE was published shortly after the release from the American Heart Association/American Diabetes Association of a Scientific Statement on T1DM and CVD in which the need for developing such tools was specified as a priority line of research [2]. The three scores consider 4–10 clinical variables for classifying subjects according to their estimated risk, which might discourage their routine use. In fact, none of the aforementioned risk scores seem to be used widely in clinical practice. Consequently, from a clinical point of view, it would seem that simpler scores might be better suited to routine clinical practice.

It is well known that some patients with T1DM can also have a certain degree of insulin resistance (IR)—a condition that has been termed “double diabetes” [14,15]. Patients with double diabetes have an increased risk for CVD events. The homeostasis model of assessment for insulin resistance (HOMA-IR) [16] was developed to estimate insulin sensitivity in non-diabetic individuals and those with non-insulin-dependent diabetes. In contrast to healthy subjects, in whom insulin secretion adapts to insulin resistance, subjects with T1DM lack endogenous insulin secretion, and therefore measurements of insulin concentrations by immunoassays or by insulin resistance indices such as HOMA-IR are not helpful in assessing insulin sensitivity [17,18]. Since 2000, several equations have been developed and validated against data from euglycemic-hyperinsulinemic clamp tests (the gold standard) to estimate whole-body insulin sensitivity or its inverse function, insulin resistance, in this population [19,20,21]. In contrast to the aforementioned scores for estimating CVD risk, these equations need fewer clinical characteristics to be run, ranging from 3 to 5, which could be an advantage for their implementation in clinical practice if they are proven to be good tools for estimating CVD risk. To the best of our knowledge, however, no previous attempt has been made to evaluate the potential relationship between the estimation of insulin sensitivity (eIS) and the estimated CVD risk assessed by risk prediction models in T1DM. Accordingly, the present study aimed to explore the potential of the estimation of IR (or its inverse function: -eIS-) for the prediction of 10-year CVD risk in adults with T1DM according to the scores of the ST1RE. For this purpose, we evaluated a group of adults with T1DM with similar clinical characteristics to those of the cohort of the Steno Diabetes Center where the ST1RE was developed.

## 2. Methods

### 2.1. Study Subjects

One hundred and seventy-nine patients aged 18–65 years, with T1DM of at least 10 years duration and without established CVD (CAD, cerebrovascular accident, or peripheral artery disease), were included in the study. Subjects were consecutively recruited from our outpatient clinic. Exclusion criteria included the following: (i) chronic kidney disease (estimated glomerular filtration rate (CKD-EPI equation [22]) <60 mL/min/1.73 m^2^), (ii) any other acute/chronic condition associated with an inflammatory response (e.g., acute or chronic inflammatory or infectious diseases), (iii) use of anti-inflammatory drugs in the previous 6 months, (iv) malignant disease in the previous 5 years (except basal cell carcinoma), (v) hospitalization in the previous 2 months, (vi) arrhythmia (other than atrial premature complex), and (vii) pregnancy. The study protocol was approved by our hospital ethics committee (Parc Taulí Research Ethics Committee, reference number 2013563, date of approval 18/6/2013) and conducted in accordance with the Declaration of Helsinki. All subjects gave their written informed consent before participating in the study.

### 2.2. Study Design

The study methods have been previously described in detail [23]. All subjects underwent standardized clinical anamnesis and physical examination. The following information was recorded using a predefined standardized form: age, sex, diabetes duration, family history of premature CVD (defined as CVD occurring before the age of 55 in males and 65 in female first-degree relatives), physical activity (International Physical Activity Questionnaire) [24], active smoking, alcohol intake, insulin dose, and the use of any other medication. Body weight, height, and waist and hip circumferences were registered. Systolic and diastolic blood pressure (SBP and DBP, respectively) were measured and mean arterial pressure (MAP) was calculated as 1/3 SBP + 2/3 DBP. After overnight fasting, venous blood samples were taken and complete blood counts, fasting plasma glucose, HbA_1c_, creatinine, and lipid profile were determined. Hypertension was defined as BP > 140/90 mmHg [25] and/or taking antihypertensive drugs. Dyslipidemia was defined as having concentrations of total cholesterol > 200 mg/dL, triglycerides > 150 mg/dL, HDL-cholesterol < 40 mg/dL, LDL-cholesterol > 130 mg/dL [26], and/or receiving drug treatment for dyslipidemia.

#### 2.2.1. Laboratory Analyses

HbA_1c_ was determined by high-performance liquid chromatography (Menarini Diagnostics, Firenze, Italy). Total serum cholesterol, triglycerides, and HDL-cholesterol were measured using standard enzymatic methods. LDL-cholesterol was estimated with the Friedewald formula [27].

#### 2.2.2. Insulin Resistance

Two different equations previously developed and validated against euglycemic-hyperinsulinemic clamp data in two independent studies (Pittsburgh EDC Study and Coronary Artery Calcification in Type 1 Diabetes -CACTI- Study) for estimating IR in adults with T1DM were used in the present study. As both equations provide an eIS, the lower the eIS, the higher the IR. The EDC equation was initially developed in the Pittsburgh EDC Study [19] and subsequently adapted by Kilpatrick et al. for the use of HbA_1c_, instead of HbA_1_ in the DCCT/EDIC cohort [28]. The EDC equation considers glycemic control, waist-to-hip ratio (WHR) and BP (eIS-EDC = 24.31 − 12.22 × (WHR) − 3.29 × (hypertension 0 = No; 1 = Yes) − 0.57 × (HbA_1c_, %)) [28]. The CACTI equation considers waist circumference, daily insulin dose per kilogram body weight, triglycerides and DBP (eIS-CACTI = 4.1075 − 0.01299 × (waist, cm) − 1.05819 × (insulin dose, UI·kg^−1^·day^−1^) − 0.00354 × (triglycerides, mg/dL) − 0.00802 × (DBP, mmHg)) [21].

#### 2.2.3. Assessment of Microvascular Complications

Peripheral polyneuropathy was assessed through a previously described two-step protocol combining the 15-item Michigan Neuropathy Screening Instrument questionnaire and a physical examination [29]. The same ophthalmologist, who was unaware of the clinical characteristics of participants, always evaluated the presence and degree of diabetic retinopathy. Subjects were classified into the following three groups according to the degree of retinopathy: no retinopathy, non-proliferative retinopathy or proliferative retinopathy. Nephropathy was assessed by the measurement of urinary albumin/creatinine ratio. Subjects with a ratio greater than 30 mg/g [30], or previously treated with converting enzyme inhibitors or angiotensin receptor blockers (for microalbuminuria or macroalbuminuria), were classified as having diabetic nephropathy.

#### 2.2.4. Steno Type 1 Risk Engine

The clinical characteristics of subjects included in the present study were used to estimate their 10-year risk of CVD events according to the ST1RE [13], accessible at www.sdcc.dk/T1riskengine. The ST1RE considers the following clinical characteristics: age, sex, smoking habit, exercise, T1DM duration, SBP, LDL-cholesterol, HbA_1c_, estimated glomerular filtration rate (CKD-EPI equation), and micro/macroalbuminuria. Based on the obtained score, subjects were classified into 3 groups according to their risk: low (<10%; *n* = 105), moderate (10–20%; *n* = 53), and high (≥20%; *n* = 21).

### 2.3. Statistical Analyses

All data were tested for normality using the Shapiro–Wilk test. Data are presented as percentages, means (standard deviation (SD)) for normally distributed quantitative variables, or medians (interquartile ranges) for non-normally distributed quantitative variables. Non-normally distributed quantitative variables were used after performing a log_10_ transformation. One-way analysis of variance (ANOVA) or the Kruskal–Wallis test was used for comparisons between groups of normally and non-normally distributed quantitative variables, as needed. The Bonferroni procedure (parametric) and the Dunn’s test (non-parametric) were used for post hoc analyses for multiple comparisons. Spearman coefficients were calculated to assess potential correlations among variables of interest. Multivariate linear regression analyses were performed to assess the potential independent relationships between 10-year CVD risk according to the ST1RE and the eIS-EDC and the eIS-CACTI equations. We tested both eIS-EDC and eIS-CACTI for discrimination using the C-statistic from logistic regression models. The C-statistic, also known as the area under the receiver-operating characteristic (ROC) curve (AUC), is an overall measure of goodness of fit for binary outcomes. Thus, it represents the probability that a randomly selected subject who experienced the outcome will have a higher predicted probability of having the outcome occur than a randomly selected subject who did not experience the outcome. ROC curves were constructed to represent C-statistic values and the prediction of 10-year CVD risk in the ST1RE. ROC curves were plotted for both moderate/high- and high-risk groups according to either eIS-EDC or eIS-CACTI results. Subsequently, the equality between the different ROC curve areas obtained with eIS equations for each risk group was tested. The best eIS cut-off point for each equation and each risk group was selected based on the Youden Index calculation. Two-tailed *p*-values < 0.05 were considered statistically significant. The calculations and figures were made using STATA v.13.1 for Mac (StataCorp LP, College Station, TX, USA) and GraphPad Prism software v 6.0 for Mac (GraphPad Software Inc., San Diego, CA, USA).

## 3. Results

**Figure 1:**
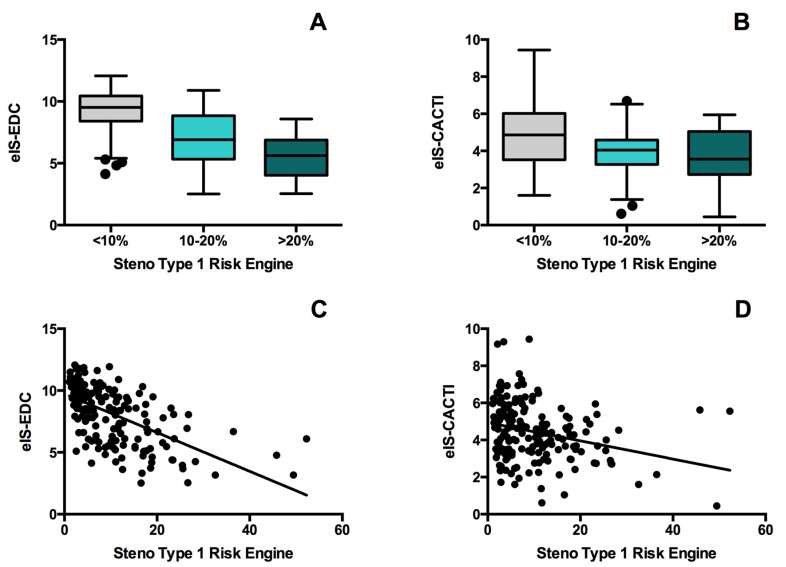
Comparison of eIS-EDC (estimated insulin sensitivity developed from the Pittsburgh Epidemiology of Diabetes Complications Study) (**A**) and eIS-CACTI (estimated insulin sensitivity developed from Coronary Artery Calcification in T1DM Study) (**B**) for the Steno Type 1 Risk Engine low (<10%), moderate (10–20%) and high-risk (≥20%) groups. Spearman correlation coefficient for the association between eIS-EDC (**C**) and eIS-CACTI (**D**) and Steno Type 1 Risk Engine risk score.

A total of 179 patients with T1DM were included in the study. Their main clinical characteristics stratified by estimated CVD risk (low, moderate, and high) are shown in Table 1. When compared with the low- and moderate-risk groups, subjects in the high-risk group were older and had a higher prevalence of hypertension and dyslipidemia. They also had worse glycemic control, longer diabetes duration, higher prevalence of microvascular complications, and greater higher body-mass index and WHR. Finally, eIS decreased as estimated CVD risk increased (Figure 1, panel A and B).

Spearman coefficients for correlations of eIS-EDC and eIS-CACTI with clinical characteristics are shown in Table 2. In univariate analyses, both eIS-EDC and eIS-CACTI correlated negatively with the ST1RE score (eIS-EDC: r = −0.635, *p* < 0.001; eIS-CACTI: r = −0.291, *p* < 0.001) (Figure 1, panel C and D). In addition, a positive correlation between eIS-EDC and eIS-CACTI was found (r = 0.487, *p* < 0.001). The correlation between the eIS-EDC with the ST1RE score was maintained (beta = −0.231; *p* = 0.011) after adjusting for the rest of traditional cardiovascular risk factors (age, gender, smoking, hypertension, dyslipidemia, and BMI). Nevertheless, the association between the eIS-CACTI with the ST1RE score was lost (beta = −0.104; *p* = 0.061).

**Figure 2:**
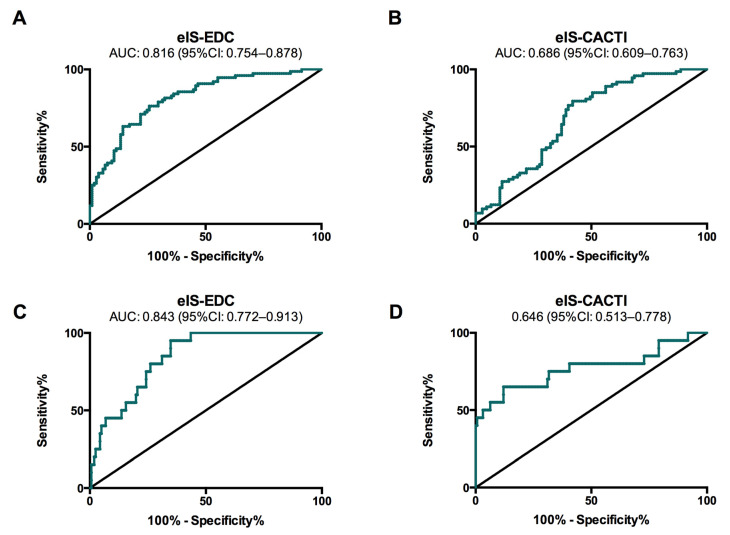
ROC curves for eIS-EDC and eIS-CACTI to identify cardiovascular risk according to the Steno Type 1 Risk Engine for moderate/high (**A**,**B**) and high-risk (**C**,**D**) groups.

To evaluate the potential of eIS for predicting the estimated CVD risk according to the ST1RE, we developed one regression model for moderate/high-risk patients and another for high-risk patients. The C-statistic of eIS-EDC was 0.816 (95% confidence interval (CI): 0.754–0.878) for predicting moderate/high risk and 0.843 (95%CI: 0.772–0.913) for predicting high risk according to the ST1RE (Figure 2, panel A and C). The best cut-off points of eIS-EDC were 8.52 mg·kg^−1^·min^−1^ (sensitivity 74%; specificity 76%) and 8.08 mg·kg^−1^·min^−1^ (sensitivity 65%; specificity 95%) for moderate/high and high risk, respectively. The C-statistic of eIS-CACTI was 0.686 (95%CI: 0.609–0.763) for predicting moderate/high risk and 0.646 (95%CI: 0.513–0.778) for predicting high risk (Figure 2, panel B and D). The best cut-off points of eIS-CACTI were 4.66 mg·kg^−1^·min^−1^ (sensitivity 58%; specificity 79%) and 3.43 mg·kg^−1^·min^−1^ (sensitivity 78%; specificity 50%) for moderate/high and high risk, respectively. The area under the ROC curve for predicting the estimated CVD risk (according to the ST1RE) was significantly higher for the eIs-EDC equation than for the eIS-CACTI equation, both for moderate/high-risk subjects (0.816 vs. 0.686; *p* = 0.001) and for high-risk subjects (0.843 vs. 0.646; *p* = 0.007).

## 4. Discussion

The present study shows that, among adults with T1DM and no previous clinical CVD, eIS is negatively associated with estimated 10-year CVD risk in the ST1RE. Additionally, we provide two cut-off points of eIS-EDC, which outperformed the eIS-CACTI equation, for detecting adults with T1DM at the highest CVD risk. These cut-off points only require three standard clinical characteristics to be calculated, which could be important for their implementation in routine clinical practice.

From a clinical perspective, the only available tool for estimating 10-year CVD risk in subjects with T1DM and no previous CVD events was the ST1RE [13]. Most of the clinical characteristics of subjects in the present study are quite similar to those of the Steno Diabetes Center cohort where the ST1RE was initially developed, supporting the use of the ST1RE in our study. These clinical characteristics were age, proportion of men, diabetes duration, body-mass index, regular exercise, proportion of people with hypertension, lipid profile, and estimated glomerular filtration rate. Because most of these characteristics influence insulin sensitivity, the negative correlation found between the scores of the ST1RE and the eIS and the good accuracy of the ROC curves was not surprising. Nevertheless, the correlation was much higher when using the eIS-EDC equation (r = −0.635) than when using the eIS-CACTI equation (r = −0.291) and was even maintained after adjusting for traditional cardiovascular risk factors. In addition, the eIS-EDC equation also outperformed eIS-CACTI in terms of C-statistic of the ROC curves, making its cut-off points superior for detecting subjects at the highest CVD risk. The differences between the two equations for eIS could be driven by how the euglycemic-hyperinsulinemic clamp studies were performed in the different studies [19,21]. Indeed, the total infused dose of insulin when validating the eIS-CACTI equation was much lower than that used when validating the eIS-EDC, which would have led to an incomplete suppression of hepatic glucose production and, consequently, to an underestimation of insulin sensitivity. This possibility is supported by the numbers given by the eIS-CACTI equation, which are much lower than those given by the eIS-EDC equation.

Simple and easy-to-use tools are essential in clinical practice for the routine measurement of variables. In this line, several equations have been developed for quantifying eIS [19,20,21], but only the two tested in the present study (eIS-EDC and eIS-CACTI) were specifically developed in adults with T1DM. Both equations require fewer clinical variables than the ST1RE. We show that the eIS-EDC performs much better than the eIS-CACTI for estimating 10-year CVD risk in the ST1RE, which would support its use in clinical practice after validation in independent cohorts.

From a pathophysiological perspective, our results are consistent with the recognized fact that IR is associated with higher CVD risk in T1DM [15]. In fact, in the prospective DCCT/EDIC cohort, some clinical characteristics clearly associated with IR, including increased HbA_1c_, BP, or body weight gain, were associated with an increase in the risk of CVD events [31,32]. In addition, and as mentioned above, most clinical characteristics associated with higher CVD risk in the ST1RE are associated with higher IR levels, such as age, smoking, T1DM duration, systolic blood pressure, HbA_1c_, estimated glomerular filtration rate, or albuminuria, supporting the potential role of IR in the prediction of CVD in T1DM.

The IR calculated from the eIS-EDC equation has previously been shown to be independently associated with a higher risk of CAD events in the Pittsburgh EDC Study [33], a higher risk of CVD events in the DCCT/EDIC Study [28], with preclinical carotid atherosclerosis [34] and even a higher risk of silent myocardial ischemia in a small cross-sectional study [35]. Likewise, the IR calculated from the eIS-CACTI equation has been independently associated with a higher risk of coronary artery calcifications [36,37]. Indeed, it has been suggested that IR could be even more important than HbA_1c_ for the prediction of CVD risk [15]. Consequently, some features have been proposed to identify people with highest IR (i.e., those with so-called double diabetes), such as the need for higher doses of insulin with progressive central obesity development, the presence of family history of type 2 diabetes, the presence of hypertension, and relatively lower levels of HDL-cholesterol or lower values for eIS.

There are some limitations in the present study that are worthy of mention. First, the study was small and cross-sectional, and a larger and prospective cohort with CVD outcomes would have been the ideal design. Instead, we took advantage of the clinical characteristics of our study subjects for estimating their 10-year CVD according to the ST1RE. Second, the study used equations for eIS previously validated against the euglycemic-hyperinsulinemic clamp, and it would have been better to develop such a type of equation in a subgroup of the evaluated subjects. Nevertheless, the negative correlation between eIS and CVD risk was found with both equations used for eIS calculations, which supports the reported results. Last but not least, the present results need to be confirmed in larger groups of adults with T1DM, especially in prospective cohorts of subjects with T1DM.

## 5. Conclusions

In conclusion, we show that eIS is negatively associated with estimated 10-year CVD risk according to the ST1RE in people with T1DM and no previous clinical CVD. In addition, we provide two cut-off points of eIS-EDC that could be of interest in routine clinical practice as only three routine clinical characteristics need to be calculated (HA1c, WHR, and hypertension). We are aware that more studies are warranted to confirm these results, especially in large prospective cohorts of people with T1DM in which CVD outcomes are recorded in a pre-specified manner.

## Figures and Tables

**Table 1:** Clinical and metabolic characteristics of patients with type 1 diabetes stratified by 10-year cardiovascular disease (CVD) risk according to the Steno Type 1 Risk Engine.

	Whole Population (*n* = 179)	Low-Risk (*n* = 105)	Moderate-Risk (*n* = 53)	High-Risk (*n* = 21)	*p* for Trend
**Clinical Characteristics**					
Age (Years)	41.2 (13.1)	32.5 (8.3)	50.8 (6.0) *	60.7 (6.6) ^†,‡^	<0.001
Sex (Male/Female), *n*	91/88	52/53	29/24	10/11	NS
Current Smokers, *n* (%)	57 (31.8)	31.0 (29.5)	21 (39.6) *	5 (23.8)	0.012
Regular Exercise, *n* (%)	142 (79.3)	86 (81.9)	40 (75.5)	16 (76.2)	0.597
Family History of Premature CVD, *n* (%)	16 (8.9)	7 (6.7)	6 (11.3)	3 (14.3)	NS
Family History of T2DM, *n* (%)	37 (20.7)	16 (15.2)	15 (28.3)	6 (28.6)	NS
Hypertension, *n* (%)	49 (27.4)	15 (14.3)	20 (33.7) *	14 (66.7) ^†,‡^	<0.001
Dyslipidemia, *n* (%)	98 (54.6)	40 (38.1)	40 (75.5) *	18 (85.7) ^†^	<0.001
**Diabetes**					
Diabetes Duration (years)	16 (12–23)	14 (20–22)	18 (15–27) *	20 (15–29) ^†^	<0.001
Total Insulin Doses (UI/kg·day)	0.6 (0.5–0.8)	0.6 (0.5–0.8)	0.7 (0.6–0.8)	0.6 (0.5–0.7)	NS
Microvascular Complications, *n* (%)	68 (38.4)	28 (27.2)	23 (43.4)	18 (81.0) ^†,‡^	<0.001
Retinopathy, *n* (%)					NS
None, *n* (%)	138 (77.1)	86 (81.9)	40 (75.5)	12 (57.1)	
Non-Proliferative, *n* (%)	20 (11.2)	9 (8.6)	6 (11.3)	5 (23.8)	
Proliferative, *n* (%)	21 (11.7)	10 (9.5)	7 (13.2)	4 (19.1)	
Nephropathy, *n* (%)	41 (23.2)	14 (13.6)	15 (28.3)	12 (57.1) ^†,‡^	<0.001
Neuropathy, *n* (%)	7 (3.9)	1 (1.0)	3 (5.7)	3 (14.3) ^†^	0.011
**Anthropometric Measurements**					
Weight (kg)	71.7 (13.0)	69.8 (12.4)	75.2 (14.3) *	72.0 (10.7)	0.045
BMI (kg/m^2^)	25.4 (3.7)	24.3 (3.2)	26.6 (3.8) *	27.8 (4.4) ^†^	<0.001
Waist-to-hip ratio	0.88 (0.81–0.94)	0.84 (0.77–0.90)	0.93 (0.86–0.99) *	0.94 (0.90–0.98) ^†^	<0.001
**Blood Pressure**					
SBP (mmHg)	125.6 (12.1)	121.8 (11.0)	128.8 (11.2) *	136.9 (10.7) ^†,‡^	<0.001
DBP (mmHg)	72.0 (8.9)	70.1 (8.2)	74.4 (8.7) *	75.7 (10.1) ^†^	0.002
MAP (mmHg)	89.9 (9.1)	87.3 (8.4)	92.5 (8.5)	96.1 (9.6)	<0.001
**Laboratory Parameters**					
Fasting Plasma Glucose (mmol/L)	8.2 (3.8)	7.8 (3.5)	8.4 (3.8)	9.5 (4.5)	NS
HbA_1c_ (%)	7.8 (1.0)	7.6 (1.0)	8.0 (1.0)	8.5 (1.1) ^†^	<0.001
HbA_1c_ (mmoL/moL)	61.8 (11.4)	59.2 (11.0)	63.7 (10.5)	69.9 (11.6)	
Urinary ACR (mg/g)	4.7 (2.7–10.6)	4.1 (2.4–7.7)	6.1 (3.0–9.8)	14.0 (5.3–54.0) ^†,‡^	<0.001
eGFR (mL·min^−1^·1.73m^−2^)	103.4 (91.2–113.6)	108.7 (05.0–117.7)	99.2 (91.8–104.0) *	83.2 (73.3–93.6) ^†,‡^	<0.001
Total Cholesterol (mg/dL)	177.9 (158.5–201.1)	174.0 (154.7–197.2)	181.7 (166.3–201.1)	197.2 (170.1–224.3)	NS
HDL-Cholesterol (mg/dL)	65.7 (50.3–77.3)	61.9 (50.3–73.5)	65.7 (54.1–81.2)	65.7 (58.0–85.1) ^†^	0.40
LDL-Cholesterol (mg/dL)	96.7 (81.2–112.1)	96.7 (85.1–112.1)	92.8 (81.2–108.3)	100.5 (88.9–119.9)	NS
Triglycerides (mg/dL)	64.6 (53.1–79.7)	63.8 (47.8–77.9)	64.7 (55.8–79.7)	67.3 (60.2–97.4)	NS
***Estimated Insulin Sensitivity***					
eIS-EDC (mg·kg^−1^·min^−1^)	8.6 (6.1–10.0)	9.5 (8.4–10.4)	6.9 (5.4–8.8) *	5.6 (4.1–6.8) ^†,‡^	<0.001
eIS-CACTI (mg·kg^−1^·min^−1^)	4.4 (3.4–5.5)	4.9 (3.5–6.0)	4.0 (3.3–4.6) *	3.6 (2.7–5.0) ^†^	<0.001

Data are given as percentages, mean (SD) or median (interquartile range). CVD: cardiovascular disease. T2DM: type 2 diabetes. BMI: body mass index. WHR: waist-to-hip ratio. SBP: systolic blood pressure. DBP: diastolic blood pressure. MAP: mean arterial pressure. ACR: urinary albumin to creatinine ratio. eGFR: estimated glomerular filtration rate. HDL: high-density lipoprotein. LDL: low-density lipoprotein. eIS-EDC: estimated insulin sensitivity developed from the Pittsburgh Epidemiology of Diabetes Complications Study. eIS-CACTI: estimated insulin sensitivity developed from Coronary Artery Calcification in T1DM Study.* *p* < 0.05 for moderate-risk compared with low-risk; ^†^ *p* < 0.05 for high-risk compared with low-risk; and ^‡^ *p* < 0.05 for high-risk compared with moderate-risk.

**Table 2:** Spearmen coefficients (rho) for correlations of the two estimated insulin sensitivity equations with clinical factors.

	eIS-EDC		eIS-CACTI	
	*rho*	*p*	*rho*	*p*
**Clinical Characteristics**				
Age (Years)	−0.545	<0.001	−0.189	0.012
Female Sex	0.382	<0.001	0.338	<0.001
Family History of T2DM	−0.079	0.296	−0.082	0.275
Hypertension	−0.726	<0.001	−0.135	0.072
Dyslipidemia	−0.382	<0.001	−0.308	<0.001
**Diabetes**				
Diabetes Duration (Years)	−0.255	<0.001	−0.010	0.891
Total Insulin Doses (UI/kg·day)	−0.092	0.223	−0.744	<0.001
Microvascular Complications	−0.490	<0.001	−0.045	0.551
Retinopathy	−0.251	<0.001	−0.014	0.859
Nephropathy	−0.580	<0.001	−0.113	0.137
Peripheral Neuropathy	−0.058	0.444	−0.057	0.453
**Anthropometric Measurements**				
Weight (kg)	−0.333	<0.001	−0.543	<0.001
BMI (kg/m^2^)	−0.380	<0.001	−0.507	<0.001
WHR	−0.748	<0.001	−0.563	<0.001
***Blood Pressure***				
SBP (mmHg)	−0.435	<0.001	−0.359	<0.001
DBP (mmHg)	−0.422	<0.001	−0.382	<0.001
**Laboratory Parameters**				
HbA_1c_ (%)	−0.354	<0.001	−0.280	<0.001
Urinary ACR (mg/g)	−0.125	0.095	0.048	0.523
Total Cholesterol (mg/dL)	0.002	0.980	−0.020	0.824
HDL-Cholesterol (mg/dL)	0.156	0.078	0.365	<0.001
LDL-Cholesterol (mg/dL)	−0.049	0.583	−0.100	0.259
Triglycerides (mg/dL)	−0.207	0.019	−0.550	<0.001
**Steno Type 1 Risk Engine**				
ST1RE Score	−0.635	<0.001	−0.291	<0.001
**Estimated Insulin Sensitivity**				
eIS-EDC (mg·kg^−1^·min^−1^)	-	-	0.487	<0.001
eIS-CACTI (mg·kg^−1^·min^−1^)	0.487	<0.001	-	-

T2DM: type 2 diabetes mellitus. BMI: body mass index. WHR: waist-to-hip ratio. SBP: systolic blood pressure. DBP: diastolic blood pressure. ACR: urinary albumin to creatinine ratio. ST1RE: Steno Type 1 Risk Engine. eIS-EDC: estimated insulin sensitivity developed from the Pittsburgh Epidemiology of Diabetes Complications Study. eIS-CACTI: estimated insulin sensitivity developed from Coronary Artery Calcification in T1DM Study.
